# The effect of grit on L2 willingness to communicate among Chinese junior high school students: an analysis of the differential roles of foreign language enjoyment and anxiety

**DOI:** 10.3389/fpsyg.2024.1468464

**Published:** 2024-10-11

**Authors:** Gengchun Li

**Affiliations:** School of Foreign Languages, Taizhou University, Taizhou, China

**Keywords:** grit, foreign language enjoyment (FLE), foreign language anxiety (FLA), willingness to communicate (WTC), Chinese junior high school students

## Abstract

With the burgeoning research on positive psychology, grit has gradually attracted scholarly attention in the field of L2 acquisition, and it has been shown to be a significant positive predictor of L2 achievements and outcomes. However, despite being an important factor in L2 acquisition, grit has rarely been incorporated as a research variable in prior research on willingness to communicate in a second language (L2 WTC), especially among Chinese teenagers who find themselves in a foundational stage of developing their L2 communicative competence. Based on a survey among 238 Chinese junior high school students, this study analyzed the effect of their grit on their L2 WTC, and explored the differential roles of foreign language enjoyment (FLE) and anxiety (FLA) in their interactions. The results revealed that: (1) perseverance of effort (PE), consistency of interest (CI) and FLE had significantly positive correlations with L2 WTC, whereas FLA was found to have a significantly negative correlation with L2 WTC; (2) PE and CI served as significant positive predictors of L2 WTC; (3) FLE mediated the interactions between PE and L2 WTC and between CI and L2 WTC, while the mediating effects of FLA were found to be statistically non-significant. The findings can provide theoretical implications for furthering L2 WTC research, as well as practical reference for its development among Chinese junior high school students.

## Introduction

The Chinese “Compulsory Education English Curriculum Standards (2022 Edition)” points out that language proficiency is a fundamental element of core literacy, referring specifically to the abilities to understand others and express oneself on relevant topics in given contexts by employing linguistic and non-linguistic knowledge. Against this backdrop, enhancing students’ abilities to express themselves in English has become one of the primary goals for English teaching in compulsory education in China. With the in-depth research on and practice of such teaching methods as group cooperative learning and the communicative approach, the issue of how to better develop students’ communicative competence has become a significant topic of concern for primary and secondary school English teachers in China. Researchers have identified that one of the key factors in promoting the development of communicative competence is learners’ willingness to communicate (WTC) (e.g., Chen et al., 2022; Wei et al., 2022). Therefore, how to enhance learners’ WTC in a second language (L2 WTC) has garnered much interest from both academics and practitioners in recent years. In its general sense, WTC pertains to an individual’s inclination to initiate oral communication with others (McCroskey and Baer, 1985), and due to its close ties with the process of L2 acquisition (e.g., Chen et al., 2022; Elahi Shirvan et al., 2019), has garnered considerable attention and interest from L2 researchers and educators (e.g., Shi and Fan, 2022; Yu, 2018). While existing research on L2 WTC has largely focused on university students in a range of cultural contexts, studies among Chinese junior high school students are relatively scarce, especially with regard to the relationship between L2 WTC and grit.

Grit, which is understood as the “mental stamina necessary to pursue long-term goals despite challenges and obstacles” (Mikami, 2024: 274) and which has been suggested to be “one of the determinants in outstanding performance across a wide range of human activities and individual pursuits” (Duckworth, 2016; cited in Lan et al., 2021: 3), may play an important role in fostering the L2 WTC among Chinese teenager students. That said, it remains unclear whether grit would exert a significant influence upon their inclination to commence oral communication with others in the L2.

Besides, L2 emotions (e.g., enjoyment and anxiety), as an important part of learners’ affective factors in L2 acquisition, could have an impact upon learners’ cognitive information processing, learning interest, behavior and engagement, self-regulation, etc., and subsequently upon their L2 learning outcomes (Shao et al., 2019). Indeed, previous studies have found a close relationship between L2 emotions such as foreign language enjoyment (FLE) and anxiety (FLA) and L2 acquisitional outcomes (e.g., Dewaele and Alfawzan, 2018; Dewey et al., 2018; Li et al., 2019; Wei et al., 2019), yet the findings remain inconsistent with regard to the roles of FLE and FLA, with some studies suggesting higher correlation or predictive power of FLE than that of FLA (e.g., Dewaele and Alfawzan, 2018; Wei et al., 2019), and other studies suggesting the other way round (e.g., Dewey et al., 2018; Li et al., 2019).

Most relevant to the current study is the research conducted by Wang (2023), which explored the influence of grit on L2 WTC, as well as the mediating effects of FLE and FLA, among a sample of 103 English major sophomores in China. One of the results of this study was that both FLE and FLA played mediating roles in the relationship between grit and L2 WTC, with stronger effect of FLE compared to FLA. To the best of our knowledge, this was the only study thus far that tried to examine the direct effect of grit, and the indirect effect thereof through the mediation of FLE and FLA, on L2 WTC in a Chinese English-as-a-foreign-language (EFL) context. However, it is still unclear whether the results obtained from its sample of undergraduate English majors (*N* = 103) could apply to junior high school students, as these two groups of students differ in a range of aspects that might influence the interrelationships among the variables under study. Considering the relevance of FLE and FLA to, and the different arguments regarding their roles in, L2 achievements and outcomes, more research on them in terms of their roles in L2 WTC is warranted. Therefore, this study tries to contribute to the ongoing discussion by focusing on grit and its influence on L2 WTC among Chinese junior high school students. Concurrently, it explores the specific roles of FLE and FLA in this relationship.

## Literature review

### Grit and L2 WTC

Grit is a concept introduced by Duckworth et al. (2007: 1087) who defined it as “perseverance and passion for long-term goals.” It is generally perceived to involve two dimensions—perseverance of effort (PE) and consistency of interest (CI) (e.g., Duckworth et al., 2007; Duckworth and Quinn, 2009). PE denotes an individual’s ability to maintain efforts in pursuing long-term goals despite adversities and failures, while CI refers to the capacity to sustain interest and enthusiasm in achieving these goals despite obstacles and setbacks.

The concept of WTC was first introduced by McCroskey and Baer (1985) for communication in the first language (L1), and was later introduced to the field of L2 acquisition by MacIntyre et al. (1998: 547) to refer to a learner’s “readiness to enter into discourse at a particular time with a specific person or persons, using a L2.” WTC serves as a prerequisite for any communicative behavior, and plays an important role in facilitating learners’ success in L2 acquisition, as it allows students to cultivate their communicative competence (Chen et al., 2022) and proceed with their L2 acquisition process effectively (Elahi Shirvan et al., 2019).

Since WTC has been found to be positively correlated with frequency of language use (e.g., MacIntyre and Charos, 1996; Yashima et al., 2004), and learners with higher WTC are more likely to master an additional language (e.g., MacIntyre et al., 2003), the level of L2 WTC is generally considered an important indicator of the success of L2 teaching and acquisition (MacIntyre et al., 1998). In view of this close connection of WTC with the process and outcome of L2 acquisition, a bulk of research has sought to validate potential predictors of WTC in an L2 context. Among these studies, the recurring variables have been learning motivation, especially under the L2 motivational self system framework (e.g., Ebn-Abbasi et al., 2022; Lan et al., 2021; Peng and Woodrow, 2010; Zhang et al., 2022; Zhang and Dai, 2024); language mindset (e.g., Sadoughi and Hejazi, 2024; Wang H. et al., 2021; Wei and Chen, 2022; Zarrinabadi et al., 2021); emotional intelligence (e.g., Dastgoshadeh and Javanmardi, 2021; Vahedi and Fatemi, 2016; Wei et al., 2022; Zou and Park, 2024); class social climate/classroom environment (e.g., Peng and Woodrow, 2010; Wang H. et al., 2021; Wang et al., 2024); language attitude (e.g., Tannenbaum and Tahar, 2008), international posture (e.g., Mystkowska-Wiertelak and Pietrzykowska, 2011; Peng, 2015), global perspective (e.g., Xiao and Qiu, 2022), among others.

Comparatively speaking, the personality factor of grit within WTC has only recently attracted scholarly attention and interest, with a few studies focusing on, for instance, grit, confidence, and motivation as predictors of L2 WTC in an Indonesian EFL context (Lee and Drajati, 2019), ideal L2 self and growth language mindset as parallel mediators in the relationship between grit and L2 WTC among Iranian EFL learners (Ebn-Abbasi et al., 2024), and FLA and/or FLE as mediators in the relationship between grit and L2 WTC in a Chinese EFL context (Wang, 2023; Yang et al., 2024). Targeting at university students across different cultural contexts, these studies have all revealed the direct predictive power of grit as well as the mediating effects of a number of other variables. However, a noticeable gap in the current literature is the lack of due attention to Chinese junior high school students, a population that finds themselves in the foundational stage of developing their communicative competence, and for whom the profiles of their grit and L2 WTC, as well as their relationships might be different from those of their college counterparts, as it was found in prior studies that age was positively associated with grit (e.g., Credé et al., 2017; Kwon, 2021; Vainio and Daukantaite, 2016), and had an important influence on L2 WTC (e.g., Cheng and Xu, 2022; Li and Li, 2022). It is therefore with this consideration that this study sets out to explore the influence of grit on L2 WTC among Chinese junior high students in an effort to contribute to the ongoing discussions regarding the role of this personality trait in a highly desirable goal of L2 education, *viz.* L2 WTC (Sadoughi and Hejazi, 2024).

### FLE, FLA, and L2 WTC

In recent years, the emergence and rapid development of positive psychology have led to burgeoning research on positive emotions in language education (see Wang Y. et al., 2021). This change has urged scholars to shift their focus from negative emotions such as boredom, anxiety, and burnout to both positive and negative emotions in L2 teaching and learning (Shafiee Rad and Jafarpour, 2023). Besides anxiety, language learners and users have reported a variety of emotions in different contexts, and among them, FLE and FLA are the two most prevalent classroom emotions (Li and Xu, 2019).

FLE refers to the feelings of joy, pleasure, and happiness experienced in classroom settings (Goetz et al., 2006). It arises from the sense of achievement and reward that one experiences when breaking through their own limitations in novel and challenging environments (Dewaele and MacIntyre, 2014, 2016). FLA, on the other hand, differs from general anxiety and specifically refers to the complex self-perceptions, beliefs, feelings, and behaviors related to foreign language learning, including communication apprehension, test anxiety, and fear of negative evaluation (Lou et al., 2024). A large number of studies have found FLE and FLA to exhibit a clear triangular relationship with L2 achievement, with a significantly positive relationship between FLE and L2 achievement, and significantly negative relationships between FLA and L2 achievement, and between FLE and FLA (see Li and Xu, 2019). However, regarding the role of FLA specifically, some qualitative studies have found that moderate levels of FLA, in fact, are conducive to L2 learning, as facilitating anxiety can have a positive and motivating influence on students (see Yu, 2022: 2). Besides, other studies have found no correlation between FLA and L2 achievement (e.g., Zhang, 2013). For example, focusing on FLA in specific language skills, it has been found that FL speaking anxiety was not significantly related to speaking proficiency, FLA did not significantly predict writing achievement, and that test anxiety did not significantly influence listening test performance in a foreign language (see Yu, 2024: 4).

Two of the most cited theories, i.e., Broaden-and-Build theory (Fredrickson, 2003) and Control-Value theory (Pekrun, 2006), lie at the core of the theories in research on L2 emotions. For one thing, the Broaden-and-Build theory emphasizes positive emotions (such as enjoyment) because they would expand an individual’s thought-action repertoire and build learners’ resilience and personal resources. Conversely, negative emotions (such as anxiety) reduce personal resources (Fredrickson, 2001). As a key component of this theory, the Undoing Hypothesis suggests that cultivating positive emotions can serve as a preventative mechanism against the long-term deleterious effects of negative emotions on health and well-being. In simpler terms, positive emotions can buffer the negative effects of negative emotions. For another, the Control-Value theory provides a framework for understanding the complex interplay between emotions, motivation, and academic achievement. The three-dimensional framework of this theory advocates defining academic emotions from three dimensions: valence (positive or negative), activation (high or low), and object focus (learning processes or performance outcomes). The valence dimension refers to the positive or negative quality of an emotion. It can be divided into positive emotions (such as joy, excitement, and pride), which enhance motivation and encourage engagement in learning, and negative emotions (such as anxiety, boredom, and frustration), which can hinder motivation and lead to disengagement and avoidance behaviors. The activation dimension pertains to the degree of arousal associated with an emotion. It distinguishes between high and low activation. High-activation emotions are characterized by intense and energetic feelings. For instance, excitement and anxiety can both cause heightened alertness and a strong physiological response. Low-activation emotions are calm and less intense, such as contentment or boredom, which might lead to passivity in learning situations. The object focus dimension relates to the source or focus of the emotion, distinguishing between emotions directed at either learning processes or performance outcomes. The former type of emotions arises from engagement in learning activities, such as interest in a new idea or frustration with a difficult concept. The latter type is tied to the results of academic performance, such as pride in achievement or shame after failure. For example, enjoyment is a positive, high-activation, process-oriented emotion, while anxiety is a negative, high-activation, outcome-oriented emotion. This theory further emphasizes that positive academic emotions facilitate learners’ cognition (such as self-regulation), motivation, and behaviors (such as learning strategies and engagement), thereby enhancing their academic achievement. Conversely, negative academic emotions constrain learners’ cognition, motivation, and behaviors, ultimately diminishing their academic achievement. Furthermore, there exists a bidirectional and dynamic relationship among academic emotions, learning processes, and academic achievement (Li and Han, 2022), which means that the academic emotions of individuals will have an influence on their learning processes, which will then exert an impact on their academic achievement, and vice versa, suggesting the pivotal role of academic emotions in learning on the one hand, and the mutual influence between academic emotions and academic achievement on the other.

Given the basic assumptions of these two theories, it has often been postulated that L2 emotions (e.g., FLE and FLA) could have significant correlations with and predict L2 WTC (e.g., Lee, 2020; Lee and Lee, 2019; Wang, 2023). Therefore, a range of studies have looked at the mediating roles of L2 emotions in the prediction of L2 WTC. Wang H. et al. (2021) investigated the effects of class social climate, language mindset, and L2 emotions (enjoyment, pride, anxiety, boredom) in predicting in-class and out-class L2 WTC among 811 Chinese college EFL learners. The results showed that the influence of the class social climate on in-class L2 WTC was fully mediated by the four emotions, while the effect of language mindset on in-class L2 WTC was fully mediated by enjoyment, pride, and boredom. Li et al. (2022) examined the relationships between classroom environment, L2 emotions (enjoyment, anxiety, boredom), and L2 WTC among 2,268 Chinese college EFL students. The results revealed that all the three L2 emotions simultaneously played mediating roles in the relationship between classroom environment and L2 WTC, with enjoyment having the largest mediating effect followed by anxiety and boredom. Wang (2023) explored the influence of L2 grit on L2 WTC and the mediating roles of FLE and FLA in this relationship among 103 Chinese college EFL students. The results indicated that PE had a direct predictive effect on L2 WTC, and both PE and CI indirectly influenced L2 WTC through the mediation of FLE and FLA. Therefore, L2 teachers should enhance students’ L2 WTC by strengthening their grittiness in L2 learning, and this can be achieved through adopting the strategies suggested by Hwang and Nam (2021), which have been proved to be effective among L2 learners (e.g., Shafiee Rad and Jafarpour, 2023), including teaching grit, reflecting on past failures, growth mindset, goal setting, deliberate practice, media use, emotional regulation, and interest discovery and maintenance. Moreover, during classroom teaching activities, L2 teachers should create a favorable language learning atmosphere that enables students to experience positive emotions, minimizing the negative impact of negative emotions as much as possible, thereby encouraging students to actively interact in the L2, and subsequently enhancing their L2 communicative skills.

Thus far, emotional variables (e.g., enjoyment, anxiety, boredom, pride, confidence) have mostly been treated as mediating factors between variables such as motivation, language mindsets, emotional intelligence, class social climate and L2 WTC. These studies utilized statistical analytic methods such as structural equation modeling or regression analysis to analyze the mediating roles of different L2 emotions in an effort to find the direct and indirect effects of external and internal variables on L2 WTC. However, among these studies, there were only a few attempting to explore the mediating roles of FLE and FLA in the relationship between grit and L2 WTC (e.g., Wang, 2023; Yang et al., 2024), and it was hypothesized that grit, as an internal variable of L2 emotions (Li and Dewaele, 2021), would have a close connection with FLE and FLA such that grittier students would have higher levels of FLE and lower levels of FLA, and accordingly higher levels of L2 WTC. Currently, there is a lack of clear understanding regarding the underlying mechanisms of these two variables in the link between grit and L2 WTC among Chinese teenagers. A question that naturally arises is: do grittier Chinese junior high school students also have higher levels of FLE, and lower levels of FLA, and does this translate into higher willingness to communicate in the L2?

For this particular student population, there are a number of interesting issues to explore. First of all, it was reported by both meta-analytical (e.g., Credé et al., 2017) and empirical studies (e.g., Kwon, 2021; Vainio and Daukantaite, 2016) that age was positively associated with grit. Does this mean that for younger students their levels of grit would be lower compared to older students? Secondly, it was found by previous studies that age had an important influence on students’ L2 WTC (e.g., Cheng and Xu, 2022; Li and Li, 2022). For example, Li and Li (2022), in their sample of 1,502 Chinese university students, found that students’ age was negatively related to L2 WTC in meaning-focused activities. Again, does this also mean that for Chinese junior high school students their levels of L2 WTC would be higher compared to college students? Moreover, as pointed out by Dewaele and MacIntyre (2014), Asian students tended to report lower levels of FLE and higher levels of FLA, and therefore it remains to be seen whether Chinese junior high school students indeed have the same pattern of emotional levels as suggested, and if so, whether this emotional pattern would make a difference on their roles in the relationship between grit and L2 WTC among Chinese young students.

## Research questions

As noted earlier, grit has been found to be an important predictor of success in both academic and non-academic domains (Teimouri et al., 2022), and FLE and FLA have been shown to be significantly correlated with L2 achievement (Li and Xu, 2019). Besides, both the levels of grit (e.g., Credé et al., 2017; Kwon, 2021; Vainio and Daukantaite, 2016) and L2 WTC (e.g., Cheng and Xu, 2022; Li and Li, 2022) have been revealed to be linked to students’ age in different ways. Moreover, the levels of FLE and FLA have also been argued to be culturally dependent. Given these results or possibilities, it is interesting to know how these variables will work synergistically in influencing the L2 WTC among Chinese junior high school students. Thus, this study tries to answer the following research questions:

*Q1*: What are the general profiles of Chinese junior high school students’ grit, FLE, FLA, and L2 WTC?

*Q2*: What are the interrelationships among Chinese junior high school students’ grit, FLE, FLA, and L2 WTC?

*Q3*: Does Chinese junior high school students’ grit directly predict their L2 WTC?

*Q4*: Does Chinese junior high school students’ grit indirectly influence their L2 WTC through the mediation of FLE and FLA?

## Research design

### Participants

Based on convenience sampling, 250 students were selected as participants from the first and second grades of a junior high school in a central city in Jiangsu Province, China. With the informed consent of their teachers and the students themselves, 250 paper questionnaires were distributed in the classrooms during students’ recess in school. After excluding invalid questionnaires (i.e., those with incomplete answers or showing a regular pattern of response such as choosing the same option from beginning to end), a total of 238 valid questionnaires were obtained, with an effective rate of 95.20%. The participants’ ages ranged from 12 to 15 years (*M* = 13.508 years, SD = 0.697), with 126 male students (52.94%) and 112 female students (47.06%). The participants reported their years of English learning between 2 to 9 years (*M* = 5.584 years, SD = 1.275).

### Research instruments

Based on previous research (as described below), four questionnaires were developed for this study, all using a Likert five-point scale format. To ensure participants’ accurate comprehension, all questionnaire items were presented in Chinese.

#### Grit scale

Among the available validated grit scales targeted specifically at L2 domain (see Liu et al., 2021), two scales were consulted for reference (Alamer, 2021; Teimouri et al., 2022). The 9-item L2 Grit scale developed by Teimouri et al. (2022) not only validated the two-factor structure of grit but also provided an effective tool for measuring L2 grit. Besides, Alamer (2021) created a 12-item L2 Grit scale, drawing inspirations from Duckworth et al.’s (2007) Grit-O scale, and Clark and Malecki’s (2019) Academic Grit scale which is specific to academic achievement and suitable for measuring grit in younger populations (sixth to eighth graders) (Sudina et al., 2021). With these considerations, the author created a new scale by combining the first 6 items of the scale developed by Alamer (2021), and the 4 reverse-coded items of the scale developed by Teimouri et al. (2022), to make it more suitable for the target respondents. The reliability and validity of the new scale were initially checked. Given the debated factorial structure of L2 grit (e.g., Credé et al., 2017; Liu et al., 2021), a confirmatory factor analysis was carried out to determine the new scale’s factorial structure, i.e., whether a two-factor or one-factor model displayed better fit to the collected data (the criteria normally adopted are: 1 < χ^2^/df < 3; GF1 > 0.9; RMSEA <0.08; RMR < 0.05; CFI > 0.9; NFI > 0.9; NNFI >0.9). The results showed that a two-factor structure comprising “perseverance of effort” and “consistency of interest” (χ^2^/df = 2.171 < 3; GFI = 0.938 > 0.9; RMSEA = 0.070 < 0.08; RMR = 0.061; CFI = 0.959 > 0.9; NFI = 0.927 > 0.9; NNFI = 0.945 > 0.9) was significantly better than a single-factor structure (χ^2^/df = 8.216 > 3; GFI = 0.775 < 0.9; RMSEA = 0.174 > 0.08; RMR = 0.159 > 0.05; CFI = 0.738 < 0.9; NFI = 0.715 < 0.9; NNFI = 0.663 < 0.9). The Cronbach’s *α* coefficients for the two sub-scales were 0.860 and 0.739, respectively, and the internal consistency reliability of the global scale was fairly satisfactory (Cronbach’s α = 0.835).

#### FLE scale

This scale was adapted from the Chinese Version of the Foreign Language Enjoyment Scale developed by Li et al. (2018) and comprised 11 items. According to the research needs, relevant expressions in the scale were modified appropriately. For example, Item 5 “It’s a positive environment” was changed to “I am surrounded by a positive environment in English learning”; Item 10 “There is a good atmosphere” was rephrased as “There is a good atmosphere of learning English in my class or school.” In this study, Cronbach’s α and KMO values of the scale were 0.855, 0.838, respectively, the Bartlett’s sphericity test was statistically significant (*p* < 0.05), the factor loadings ranged from 0.639 to 0.875, and the cumulative percentage of variance explained after rotation was 51.680%, which all indicated that the scale had good reliability and validity.

#### FLA scale

This scale was adopted from the Foreign Language Classroom Anxiety Scale developed by Dewaele and MacIntyre (2014), consisting of 8 items. In this study, Cronbach’s α coefficient of the scale was 0.801, the KMO value was 0.815, the Bartlett’s sphericity test was statistically significant (*p* < 0.05), the factor loadings ranged from 0.610 to 0.871, and the cumulative percentage of variance explained after rotation was 56.446%, indicating that the scale had good reliability and validity.

#### L2 WTC scale

This scale was taken from the WTC scale developed by Peng and Woodrow (2010) and comprised 10 items. In this study, Cronbach’s α coefficient and KMO value of this scale were 0.887, 0.872, respectively, the Bartlett’s sphericity test was statistically significant (*p* < 0.05), the factor loadings ranged from 0.549 to 0.882, and the cumulative percentage of variance explained after rotation was 66.852%, indicating good reliability and validity of this scale.

### Data analysis

Data analysis in this study was conducted using the SPSSAU (an online platform for conducting statistical analysis at https://www.spssau.com), including tests for common method bias, descriptive statistics, correlation analysis, Kruskal-Wallis test, one-way ANOVA, and mediation analysis.

## Results

### Test for common method bias

This study employed the Harman’s single-factor test to check for common method bias. Common method bias refers to the systematic error that can occur when the same method is used to collect data for different variables in research, leading to inflated relationships among those variables. This bias often arises from the reliance on a single source or method, such as self-reported surveys or questionnaires, which can influence responses and create spurious correlations. The results showed that a total of 9 factors had eigenvalues greater than 1, with the variance explained by the first factor at 28.24%, below the critical threshold of 40%. This indicates that the data in this study do not exhibit severe common method bias, and can be subjected to subsequent analysis.

### Descriptive statistics and correlation analysis

**Figure 1:**
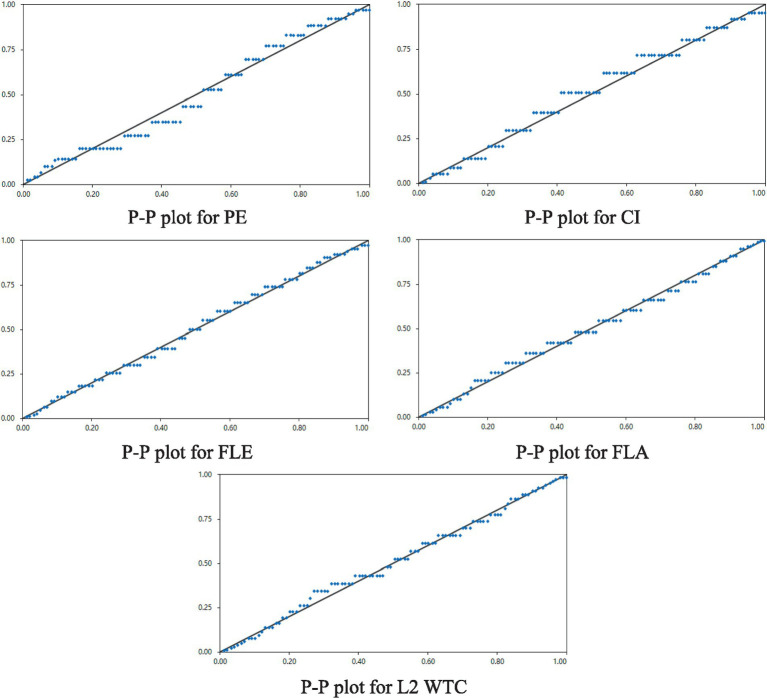
The P–P plots for all the variables.

The normality test (K-S test) results indicated that the variables FLE and FLA followed a normal distribution (*p* = 0.114, 0.052), while other variables, although not normally distributed, showed acceptable skewness values. Further checks with the P–P plots (see Figure 1) showed that the data for all variables were reasonably normally distributed, with the means at a moderate or slightly above-moderate level (in this study, we take means between 2.1–3.5 as being at a moderate level) (see Table 1).

**Table 1:** Descriptive statistics (*N* = 238).

Variables	Range	Mean	Standard deviation	Skewness	Kurtosis
PE	1–5	3.624	0.741	−0.011	−0.449
CI	1–5	3.488	0.905	−0.404	−0.280
FLE	1–5	3.641	0.703	−0.205	−0.291
FLA	1–5	3.044	0.815	−0.026	0.076
L2 WTC	1–5	3.152	0.866	−0.179	−0.108

PE, perseverance of effort; CI, consistency of interest; FLE, foreign language enjoyment; FLA, foreign language anxiety; L2 WTC, L2 willingness to communicate.

The results of the correlation analysis (see Table 2) indicated that: (1) there was a significant positive correlation between PE, CI, FLE and L2 WTC, with a significant negative correlation between FLA and L2 WTC; (2) PE was significantly positively correlated with CI and FLE, and significantly negatively correlated with FLA; (3) CI was significantly positively correlated with FLE and significantly negatively correlated with FLA; (4) FLE was significantly negatively correlated with FLA.

**Table 2:** Correlation matrix.

Variables	1	2	3	4	5
1.PE	1.000	0.414^**^	0.627^**^	−0.250^**^	0.525^**^
2. CI	0.414^**^	1.000	0.459^**^	−0.394^**^	0.374^**^
3. FLE	0.627^**^	0.459^**^	1.000	−0.312^**^	0.594^**^
4. FLA	−0.250^**^	−0.394^**^	−0.312^**^	1.000	−0.301^**^
5. L2 WTC	0.525^**^	0.374^**^	0.594^**^	−0.301^**^	1.000

1. PE: perseverance of effort; CI: consistency of interest; FLE: foreign language enjoyment; FLA: foreign language anxiety; L2 WTC: L2 willingness to communicate; 2.^**^ *p* < 0.01.

Since it was found by other researchers that age was positively associated with grit (e.g., Credé et al., 2017; Kwon, 2021) and had a significant influence on L2 WTC (Cheng and Xu, 2022; Li and Li, 2022), female students tended to be gritter, had higher levels of FLE (Wei et al., 2019) and were more willing to communicate using an L2 than male students (Cheng and Xu, 2022), and that learning experience positively predicted L2 WTC in class (Peng, 2015), one-way ANOVA or non-parametric test was run to check for their possible influence on the variables under study. Given the uneven distributions of age and years of English learning for the sample of this study, non-parametric tests were used to check for their possible influence on all the key variables. A Kruskal-Wallis test showed that age did not have a significant influence on PE (*H* = 1.417, *p* = 0.702), FLE (*H* = 4.184, *p* = 0.242), and L2 WTC (*H* = 1.886, *p* = 0.596), but had a significant influence on CI (*H* = 11.698, *p* = 0.008), and FLA (*H* = 8.360, *p* = 0.039). The same test showed that students’ years of English learning did not significantly influence PE (*H* = 5.507, *p* = 0.598), CI (*H* = 6.553, *p* = 0.477), FLE (*H* = 4.451, *p* = 0.727), FLA (*H* = 2.945, *p* = 0.890), or L2 WTC (*H* = 3.447, *p* = 0.841). Finally, the results of a one-way ANOVA showed that gender did not significantly affect all the variables in the present study, either (*p* = 0.21, 0.14, 0.31, 0.32, 0.65). Given these results, in the subsequent analyses, age will serve as a control variable for the mediation analysis.

### Analysis of the mediating effects of FLE and FLA

In this study, the Bootstrap bias-corrected method (with 5,000 samples) was employed to analyze the mediating effects of FLE and FLA in the relationship between the two dimensions of grit and L2 WTC. Specifically, the mediation analysis was conducted using the SPSSAU platform, with age as a control variable. The variables of PE, CI, FLE, FLA, and L2 WTC were entered into the model to estimate the effect sizes of the mediation and confidence intervals. The regression analysis results (see Table 3) indicated that PE and CI significantly positively predicted L2 WTC (*β* = 0.446, *p* < 0.01; *β* = 0.192, *p* < 0.01) and FLE (*β* = 0.528, *p* < 0.01; *β* = 0.242, *p* < 0.01); CI significantly negatively predicted FLA (*β* = −0.341, *p* < 0.01). When all predictor variables were simultaneously entered into the regression equation, CI and FLA did not significantly predict L2 WTC; however, PE and FLE significantly positively predicted L2 WTC (*β* = 0.229, *p* < 0.01; *β* = 0.391, *p* < 0.01).

**Table 3:** Regression analysis of the relationships among variables in the model.

Regression equation		Overall fit index			Significance of regression coefficients	
Outcome variable	Predictor variable	*R* ^2^	Adapted *R*^2^	*F*	*β*	*t*
L2 WTC	Age	0.305	0.296	34.255^**^	0.017	0.304
	PE				0.446	7.442^**^
	CI				0.192	3.176^**^
FLE	Age	0.442	0.435	61.738^**^	0.004	0.077
	PE				0.528	9.830^**^
	CI				0.242	4.453^**^
FLA	Age	0.167	0.156	15.613^**^	0.052	0.865
	PE				−0.107	−1.633
	CI				−0.341	−5.145^**^
L2 WTC	Age	0.405	0.392	31.623^**^	0.020	0.398
	PE				0.229	3.456^**^
	CI				0.064	1.051
	FLE				0.391	5.730^**^
	FLA				−0.099	−1.763

1. PE: perseverance of effort; CI: consistency of interest; FLE: foreign language enjoyment; FLA: foreign language anxiety; L2 WTC: L2 willingness to communicate; 2. ^**^ *p* < 0.01.

Results of the mediation analysis (see Table 4) indicated that: (1) FLE partially mediated the relationship between PE and L2 WTC, accounting for 46.33% of the effect size; (2) FLE fully mediated the relationship between CI and L2 WTC; (3) FLA did not show significant mediating effects in either the relationship between PE or CI and L2 WTC.

**Table 4:** Results of mediation effect test.

Pathway of mediation effect	a	b	a*b	95% Boot *CI*	c’	Conclusion
PE → FLE → L2 WTC	0.501^**^	0.483^**^	0.242	0.122 ~ 0.298	0.267^**^	Partial mediation
PE → FLA → L2 WTC	−0.118	−0.105	0.012	−0.001 ~ 0.050	0.267^**^	No significant mediation
CI → FLE → L2 WTC	0.188^**^	0.483^**^	0.091	0.042 ~ 0.171	0.061	Full mediation
CI → FLA → L2 WTC	−0.307^**^	−0.105	0.032	−0.001 ~ 0.090	0.061	No significant mediation

1. PE: perseverance of effort; CI: consistency of interest; FLE: foreign language enjoyment; FLA: foreign language anxiety; L2 WTC: L2 willingness to communicate; 2. ^**^ *p* < 0.01.

## Discussion

### Overall levels of and interrelationships among variables

Descriptive statistics revealed that Chinese junior high school students’ PE (*M* = 3.624, SD = 0.741), CI (*M* = 3.488, SD = 0.905), FLE (*M* = 3.641, SD = 0.703), FLA (*M* = 3.044, SD = 0.815), and L2 WTC (*M* = 3.152, SD = 0.866) stood at or slightly above the moderate range. When the grit construct is viewed as a whole, the level of overall grit in the present study is significantly lower than that in Wang’s (2023) study (*t* = −3.533, *p* < 0.01, Cohen’s *d* = 0.229). This may suggest the likelihood that as students get older, their grit in L2 learning may grow stronger, aligning with the findings of some researchers (e.g., Credé et al., 2017; Kwon, 2021; Vainio and Daukantaite, 2016). Besides, the level of L2 WTC was significant lower than that of Wang (2023) as well (*t* = −11.536, *p* < 0.01), which does not accord with Li and Li’s (2022) finding that students’ age is negatively correlated with their L2 WTC. Moreover, the level of FLE is significantly higher than that of FLA (*t* = 7.483, *p* < 0.01), which means that Chinese junior high school students indeed have higher levels of positive emotions than do negative emotions, not in line with the argument of Dewaele and MacIntyre (2014). However, caution should be exercised when interpreting the results of this comparison as the research participants do not study at the same educational level or in the same learning environment. Therefore, a longitudinal study probing into the changes of L2 students’ grit, FLE, FLA, or L2 WTC should better reveal the dynamic nature of these constructs.

As noted earlier, the results of the correlation analysis indicated significant positive correlations between Chinese junior high school students’ PE, CI and their L2 WTC, consistent with previous research findings (e.g., Teimouri et al., 2022; Wang, 2023). This suggests that the greater the effort and interest Chinese students invest into learning English, the more actively they engage in English interaction and communication in class, affirming that grit is an important personality factor influencing their willingness to communicate. As widely acknowledged, L2 learning is a challenging process fraught with various obstacles, setbacks, and even failures. For Chinese junior high school students, communicating in English with their teachers and classmates is also a challenging task full of uncertainty, nervousness and anxiety. Learners need to have the mental stamina to overcome challenges and obstacles, as well as the perseverance and passion required to pursue long-term goals, which is at the core of the concept of grit. Hence, the higher the level of grit among learners, the higher their psychological readiness to initiate or engage in L2 communication and interaction.

Furthermore, Chinese junior high school students’ PE and CI were significantly correlated with their levels of FLE and FLA. This indicates that learners’ personality traits influence their emotional experiences in foreign language learning. The top-down theories of subjective well-being posit that a stable personality trait, as in the case of grit (Duckworth et al., 2007), affects individuals’ interactions with and interpretations of reality, thereby impacting their subjective well-being (see Hou et al., 2022), which in turn projects onto the process of foreign language learning, affecting their emotional experiences in this process. Thus, this study found that Chinese junior high school students’ grit (*viz.* PE and CI) as a personality trait influences their emotional experiences in foreign language learning, further corroborating the subjective well-being theory.

Additionally, FLE and FLA were significantly positively and negatively correlated, respectively, with L2 WTC, aligning with previous research conclusions (e.g., Li et al., 2022; Li and Han, 2022; Wang, 2023). This suggests that the more enjoyment Chinese junior high school students experience, the lower their anxiety, the stronger their willingness to communicate, confirming that FLE and FLA, as emotional variables, are crucial factors influencing communicative willingness in the L2. As previously mentioned, WTC is a prerequisite for any communicative behavior, and the amount and quality of communicative behavior directly affects the improvement of learners’ communicative abilities. Therefore, English teachers in junior high schools in China need to closely monitor students’ emotional experiences in learning, enhance students’ FLE from multiple perspectives (e.g., from the perspective of teachers, environment, and individuals), buffer the negative impact of FLA, and consequently boost students’ willingness to communicate in the L2.

### Direct and indirect effects of grit on L2 WTC

As discussed above, the results of a multiple regression analysis revealed that Chinese junior high school students’ PE and CI both significantly and positively predicted their L2 WTC. This finding differs from some research conclusions (e.g., Lee, 2020; Wang, 2023). For example, Lee’s (2020) study found that PE on the part of junior high school, senior high school, and college students could predict their WTC, but CI could not. Wang’s (2023) study discovered that while CI among second-year English majors was positively correlated with their L2 WTC, it did not have a significant predictive effect on the latter. Indeed, some previous studies indicate that CI has less predictive power for various outcomes compared to PE or overall grit (Khajavy and Aghaee, 2024). For example, Credé et al. (2017)‘s meta-analysis found that PE had an advantage over CI or overall grit in predicting performance. Lam and Zhou’s (2022) meta-analysis showed that the relationship between PE and academic achievement was the closest, followed by overall grit and CI. This difference in results could be attributed to two factors. One is that the scales used to measure L2 grit differed across these studies. To be specific, Lee (2020) employed the Grit-S scale developed by Duckworth and Quinn (2009) to measure domain-general grit (i.e., grit not specifically targeted at the L2 domain), and Wang (2023) adopted the L2 grit scale developed by Teimouri et al. (2022), while in the present study, a new L2 scale was developed based on Alamer (2021) and Teimouri et al. (2022). Besides, the respondents chosen were different. While Wang (2023) focused on Chinese undergraduate English majors, and Lee (2020) chose to study a mixed sample of junior high school, senior high school and college students in Korea, the current study involved junior high school students in a Chinese EFL context.

Therefore, for this particular student population, both PE and CI exerted an important influence on their L2 WTC. This result may also suggest the possibility that for learners at different educational levels, PE and CI may have varying predictive power on their L2 WTC. As few studies made a comparison between learners from different educational levels, more research is needed to explore this possibility.

The results of the mediation analysis demonstrated that FLE mediated the relationship between grit and L2 WTC among Chinese junior high school students. Specifically, FLE partially mediated the relationship between PE and L2 WTC, while it fully mediated the relationship between CI and L2 WTC. Therefore, grit in Chinese junior high school students can directly influence their L2 WTC and indirectly affect it through the mediation of FLE. In other words, gritter students indeed have the mental stamina to overcome barriers, setbacks, and even failures in their L2 learning, including in learning to use their L2 to communicate with their classmates and teachers, and therefore they are more mentally prepared to initiate or engage in L2 communication. They will also have higher levels of enjoyment in L2 learning, which will further boost their willingness to communicate in the L2. Furthermore, FLA did not show a significant mediating effect in the relationship between PE or CI and L2 WTC. This result suggests that while anxiety may cause learners to feel nervous and uneasy when communicating in a foreign language, junior high school students with high levels of PE and CI will not decrease their willingness to communicate in English due to anxiety.

Based on the results of this study, English teachers in junior high schools in China are advised to adopt the following strategies (Hwang and Nam, 2021; Shafiee Rad and Jafarpour, 2023): (1) teaching the meaning, importance, and practice of grit to students, preferably by relating grit to successes in L2 learning through real cases; (2) asking students to reflect on failure experiences they had before in their L2 learning, and helping them to analyze their causes; (3) providing feedback to the students on the process instead of the outcome of L2 learning, on the efforts they have made rather than their abilities, while keeping reminding them that one’s abilities can be improved through efforts; (4) establishing high yet realistic expectations for students, for example by challenging students appropriately in their L2 learning, pushing them out of their comfort zones, while ensuring timely guidance and assistance when they face obstacles; (5) involving students’ interest in learning by helping them to understand new, complex, or difficult situations, while encouraging them to explore their personal passions through extracurricular activities and projects, fostering enduring enthusiasm for L2 learning; (6) creating a lively and enjoyable classroom climate (Wang, 2023) by building friendly relationships between the teacher and students, and among students as well, so as to enhance their levels of positive emotions.

## Conclusion

The present study focused on Chinese junior high school students, an apparently under-researched population, and utilized multiple regression analysis to investigate the predictive effect of their grit on their willingness to communicate in an L2, as well as the differential roles of their foreign language enjoyment and anxiety in this relationship. The results indicated that both perseverance of effort and consistency of interest within grit directly influenced their willingness to communicate and indirectly affected it through the mediation of foreign language enjoyment. Foreign language anxiety did not significantly mediate the relationship between grit and willingness to communicate in the L2.

This study could provide insights for English teaching in junior high schools in China. Based on the results, English teachers in junior high schools are advised to enhance students’ perseverance of effort and consistency of interest in English learning to boost their willingness to communicate, while also assigning equal importance to nurturing students’ positive emotions in English learning. In this regard, teachers are supposed to create a positive classroom atmosphere for students to experience positive emotions during classroom activities, thereby enhancing their willingness to communicate in English.

Despite these initial findings, the present study may suffer from several limitations. First of all, the sample size of this study was not large enough, and it did not involve higher-grade students. Secondly, the cross-sectional design of the study failed to keep track of the dynamic changes in grit, foreign language enjoyment and anxiety, and willingness to communicate in English, whose relationship may well change over time. Thirdly, this study focused only on enjoyment and anxiety, to the neglect of other L2 emotions such as boredom and burnout, which may also play a role in influencing students’ willingness to communicate in English. These questions could be further explored in future studies.

## Data Availability

The original contributions presented in the study are included in the article/Supplementary material, further inquiries can be directed to the corresponding author.
